# Circumscribed Morphea and Breast Asymmetry in an Adolescent

**DOI:** 10.1155/2014/418257

**Published:** 2014-01-08

**Authors:** António Augusto Fernandes Massa, Armando Manuel Simões Baptista, António Manuel Ferreira da Silva Abreu Couceiro, Eduarda Macedo Osório Morais Ferreira

**Affiliations:** ^1^Department of Dermatology, Centro Hospitalar de V.N.Gaia/Espinho, EPE, 4430 Vila Nova de Gaia, Portugal; ^2^Department of Pathology, Centro Hospitalar de V.N.Gaia/Espinho, EPE, 4430 Vila Nova de Gaia, Portugal

## Abstract

Morphea is a rare fibrosing disorder of the skin and underlying tissues. Circumscribed morphea presents with less than three discrete indurated plaques and breasts are commonly affected in women. We report the case of a 12-year-old female with a right infra-areolar, nontender, brownish patch and asymmetry of the right breast with 2 years of evolution. Skin biopsy showing thickening of the dermal collagen bundles confirmed the clinical diagnosis of morphea. After a 3-year follow-up period without progression of disease, reconstructive surgery is scheduled. Plaque morphea can involve all layers of the skin but associated breast deformity is rare. It can mimic benign and malignant breast disorders justifying the benefit for early tissue biopsy. Breast morphea generally has a good prognosis but hyperpigmentation and breast deformity in young girls have been rarely described. An early diagnosis can possibly lead to a therapeutic intervention with a different outcome, as it can be the source of severe psychological and social issues in a delicate period of development such as adolescence.

## 1. Introduction

Morphea is a rare fibrosing disorder of the skin and underlying tissues, with equal prevalence both in adults and children, female predominance, and greater prevalence in caucasians. Circumscribed morphea presents with less than three discrete indurated plaques, predominantly on the trunk, and it can be superficial or deep [1–3]. Breasts are commonly affected in women, uniformly sparing the nipples [4].

## 2. Case Presentation

We report the case of a 12-year-old female patient who was referred to our outpatient clinic for evaluation of a patch in her right breast with 2 years of evolution. Pruritus and pain were denied. No musculoskeletal, respiratory, gastrointestinal, neurologic, and vascular symptoms were present. Her past medical history was irrelevant except for asthma and there was no family history of autoimmune diseases.

**Figure 1:**
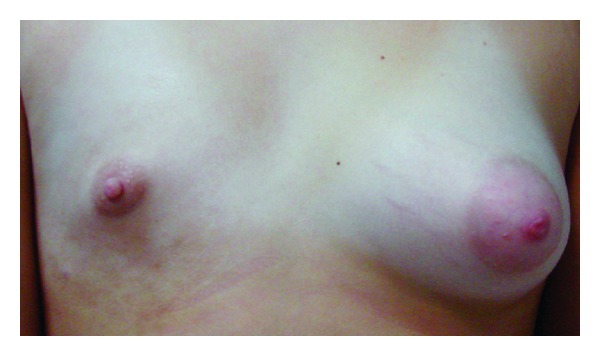
Right breast asymmetry, infra-areolar patch, with 7 cm, with nontender, brownish, hyperpigmented, and hypopigmented areas.

At the physical exam, right breast asymmetry was noted, with an infra-areolar patch with 7 by 3 cm, with nontender, brownish, hyperpigmented, and hypopigmented areas (Figure 1). There were no other relevant cutaneous lesions.

**Figure 2:**
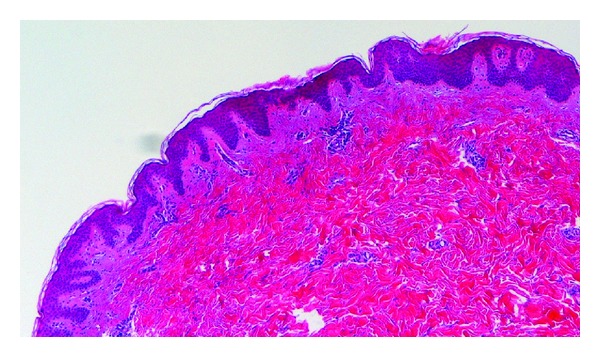
Haematoxylin and eosin—thickening of the dermal collagen bundles with a perivascular lymphohistiocytic infiltrate.

A skin biopsy was performed, showing thickening of the collagen bundles in the dermis with a perivascular lymphohistiocytic infiltrate. No eccrine glands were present (Figure 2). These histologic findings confirmed the clinical diagnosis of morphea.

Screening for systemic involvement and for *Borrelia burgdolferi*, as well as for autoimmunity was negative, including antinuclear antibodies and extractable nuclear antigens.

Breast ultrasonography showed scarce mammary tissue at the right breast. Skin and subcutaneous tissue showed no alterations, suggesting breast hypoplasia.

Although a topical corticosteroid or a topical calcineurin inhibitor could be indicated, given the absence of symptoms, functional limitations, and tenderness, and with the parents' acknowledgement, no therapeutic intervention was performed.

After a 3-year follow-up period without progression of disease, reconstructive surgery with breast augmentation is scheduled.

## 3. Discussion

Plaque Morphea can involve all layers of the skin, subcutaneous tissue, and underlying bone but associated breast deformity is rare.

Breast associated morphea can mimic benign and malignant inflammatory breast disorders, being misdiagnosed in two-thirds of the cases as inflammatory breast cancer or breast infections, justifying the benefit for early tissue biopsy in patients with unexplained breast erythema to confirm a clinical diagnosis and thus guide subsequent interventions [5].

While idiopathic morphea, all subtypes included, is an infrequent disease with a reported incidence of 2.7 per 100.000 in the United States [6, 7], breast associated morphea is a relatively common effect after radiation therapy for breast cancer, with an incidence as high as 1 in every 500 patients [8, 9]. It is usually confined to the radiated site but can also rarely occur distant to the radiation site [10].

Breast morphea generally has a good prognosis and did not result in significant disfiguration and morbidity [5, 11], but hyperpigmentation and breast deformity in young girls have been described [12–15].

It should be thought when evaluating an adolescent for breast deformity [12–15] and kept in mind as an early diagnosis that can possibly lead to a therapeutic intervention with a different outcome.

Physicians should be aware and recognize the signs of what could result in a major burden of disease, with severe adverse psychological and social implications in a delicate period of development such as adolescence.
